# Migrating a Well-Established Longitudinal Cohort Database From Oracle SQL to Research Electronic Data Entry (REDCap): Data Management Research and Design Study

**DOI:** 10.2196/44567

**Published:** 2023-05-31

**Authors:** Katharina Kusejko, Daniel Smith, Alexandra Scherrer, Paolo Paioni, Malte Kohns Vasconcelos, Karoline Aebi-Popp, Roger D Kouyos, Huldrych F Günthard, Christian R Kahlert, Swiss HIV Cohort Study and the Swiss Mother and Child HIV Cohort Study

**Affiliations:** 1 Institute of Medical Virology University of Zurich Zurich Switzerland; 2 Division of Infectious Diseases and Hospital Epidemiology University Hospital Zurich Zurich Switzerland; 3 Division of Infectious Diseases and Hospital Epidemiology University Children's Hospital Zurich Zurich Switzerland; 4 Department for Infectious Diseases and Vaccinology University of Basel Children’s Hospital Basel Switzerland; 5 Department of Infectious Diseases Inselspital Bern, University of Bern Bern Switzerland; 6 Department of Obstetrics and Gynecology Lindenhofspital Bern Switzerland; 7 Department of Infectious Diseases and Hospital Epidemiology Cantonal Hospital St Gallen St Gallen Switzerland; 8 Division of Infectious Diseases and Hospital Epidemiology Children’s Hospital of Eastern Switzerland St Gallen Switzerland; 9 See Acknowledgements

**Keywords:** REDCap, cohort study, data collection, electronic case report forms, eCRF, software, digital solution, electronic data entry, HIV

## Abstract

**Background:**

Providing user-friendly electronic data collection tools for large multicenter studies is key for obtaining high-quality research data. Research Electronic Data Capture (REDCap) is a software solution developed for setting up research databases with integrated graphical user interfaces for electronic data entry. The Swiss Mother and Child HIV Cohort Study (MoCHiV) is a longitudinal cohort study with around 2 million data entries dating back to the early 1980s. Until 2022, data collection in MoCHiV was paper-based.

**Objective:**

The objective of this study was to provide a user-friendly graphical interface for electronic data entry for physicians and study nurses reporting MoCHiV data.

**Methods:**

MoCHiV collects information on obstetric events among women living with HIV and children born to mothers living with HIV. Until 2022, MoCHiV data were stored in an Oracle SQL relational database. In this project, R and REDCap were used to develop an electronic data entry platform for MoCHiV with migration of already collected data.

**Results:**

The key steps for providing an electronic data entry option for MoCHiV were (1) design, (2) data cleaning and formatting, (3) migration and compliance, and (4) add-on features. In the first step, the database structure was defined in REDCap, including the specification of primary and foreign keys, definition of study variables, and the hierarchy of questions (termed “branching logic”). In the second step, data stored in Oracle were cleaned and formatted to adhere to the defined database structure. Systematic data checks ensured compliance to all branching logic and levels of categorical variables. REDCap-specific variables and numbering of repeated events for enabling a relational data structure in REDCap were generated using R. In the third step, data were imported to REDCap and then systematically compared to the original data. In the last step, add-on features, such as data access groups, redirections, and summary reports, were integrated to facilitate data entry in the multicenter MoCHiV study.

**Conclusions:**

By combining different software tools—Oracle SQL, R, and REDCap—and building a systematic pipeline for data cleaning, formatting, and comparing, we were able to migrate a multicenter longitudinal cohort study from Oracle SQL to REDCap. REDCap offers a flexible way for developing customized study designs, even in the case of longitudinal studies with different study arms (ie, obstetric events, women, and mother-child pairs). However, REDCap does not offer built-in tools for preprocessing large data sets before data import. Additional software is needed (eg, R) for data formatting and cleaning to achieve the predefined REDCap data structure.

## Introduction

The era of the digital transformation has reached the public health sector, with personalized medicine, machine-readable medical terminology, and fully automated data transfers being priorities for research and funding institutions [1,2]. This is also reflected in the vast efforts of researchers striving to find optimal digital solutions for clinical studies and medical data flow; for example, the search terms “digital health” and “digitalization” on PubMed have yielded exponentially growing numbers of results in the last years, with around 1500 publications since 2020. To master this new challenge, political commitment, regulatory frameworks, and funding are needed to promote the progress of digitalization in the health care sector, as also stated by the European Public Health Association [3]. Education, as well as research, is needed for optimizing the implementation of the necessary information technology (IT) infrastructure, such as automation of data flows. While data analysis experts have been well integrated in clinical research groups, the need for experts to set up databases, curate the data, and build efficient data flows has not been given equal attention [4,5]. Only recently, large funding organizations, such as the European Research Council, have integrated data management plans as mandatory, key documents for successful research projects, highlighting the importance of optimal data collection and storing processes [6,7].

To alleviate the potential lack of IT database specialists, user-friendly clinical data management software has been developed with the aim of setting up data collection tools without the need for in-depth programming or database knowledge. One example of such a software solution is Research Electronic Data Capture (REDCap), which allows the user to build electronic case report forms, monitor study progress, and extract data in a predefined format [8]. REDCap has been developed for medical research and is adherent to ethical standards, including logging tools and randomization options for clinical trials. It has been used for electronic data collection in more than 1.4 million research projects, including projects in resource-constrained environments [9,10] and for mobile data collection in outreach settings [11]. While REDCap is a robust application for setting up clinical research projects and collecting new study data, the situation is more complicated when it comes to interoperability with other database systems, where tailored solutions using additional software components are necessary. For example, the REDCap Clinical Data Interoperability Service module allows for data exchange between REDCap and electronic health records stored in clinical data warehouses, avoiding manual transcription of data [12]. Other add-on modules facilitate real-time data analysis by automatically extracting REDCap data to an external relational data base [13], migrating legacy data with the *REDLetr* module [14], and integrating a semantic annotation framework to extend the metadata of REDCap studies [15]. Pipelines using additional software, such as R, have been developed to reformat and transfer electronic health records to REDCap for multicenter patient registries [16,17]. Moreover, ways to collect clinical data via REDCap and transfer them to electronic health records (ie, the opposite direction of data flow) have been implemented by others [18].

In this project, we report on the migration of data collected in the Swiss Mother and Child HIV Cohort Study (MoCHiV) from Oracle SQL (Oracle Corp) to REDCap. MoCHiV is a multicenter longitudinal cohort study with approximately 100,000 data records dating back to the early 1980s [19-21]. Data have been prospectively collected via paper forms all over Switzerland submitted by mail to the data center of the Swiss HIV Cohort Study, where data managers transfer the data manually to the Oracle database. The main motivation for the database migration to REDCap was to provide an intuitive, user-friendly graphical user interface (GUI) for electronic data collection at the local study sites.

## Methods

### Study Design of MoCHiV

MoCHiV was formally established in 2003 by combining 2 ongoing studies (the Neonatal HIV Study and the HIV Pregnancy Study) that enrolled HIV-infected children and pregnant women living with HIV, respectively [22]. Since then, MoCHiV has been a substudy of the Swiss HIV Cohort Study (SHCS), a multicenter longitudinal cohort study enrolling adults living with HIV in Switzerland since 1988 [19]. MoCHiV collects information about pregnancies and deliveries of women living with HIV, along with data on regular follow-up visits within the SHCS after delivery. Children living with HIV are followed until the age of 18 years and are then transferred to the SHCS. HIV-exposed uninfected children are followed until the age of 5 years.

### Ethical Considerations

MoCHiV was approved by all local ethics committees of the participating centers [23]: the Ethikkommission beider Basel (688), Kantonale Ethikkommission Bern (21/88), Comité départemental d'éthique des spécialités médicales et de médecine communautaire et de premier recours, Hôpitaux Universitaires de Genève (01–142); Commission cantonale d'éthique de la recherche sur l'être humain, Canton de Vaud (131/01), Comitato etico cantonale, Repubblica e Cantone Ticino (CE 813); Ethikkommission des Kantons St. Gallen (EKSG 12/003), and Kantonale Ethikkommission Zürich (KEK-ZH-NR: EK-793). Written informed consent was obtained for all adult participants and parents of pediatric patients. The patients were not compensated for participating in this study. The collected data are deidentified and stored on a secured server of the University of Zurich, Switzerland. Use of data for research purposes must be approved by the Scientific Board of the SHCS/MoCHiV and requires a data transfer agreement to ensure protection of the collected data.

### Technical Tools for Preparing Migration

Until 2022, MoCHiV data were stored in an Oracle database (most recently version 19) with a GUI for data entry designed with Oracle Application Express [24]. The new electronic data entry system presented in this paper was designed using REDCap (version 12.5.10; Vanderbilt University) [8]. R (version 4.1.0; R Studio) was used for data cleaning and reformatting. SHCS data are stored in an Oracle database with a GUI developed with the Django framework (Django Software Foundation). Every application (REDCap, Oracle, and Django) was set up in a Docker (Docker Inc) repository on a server hosted by the University of Zurich with automated daily back-ups.

## Results

### Overview of the Migration Process

**Figure 1:**
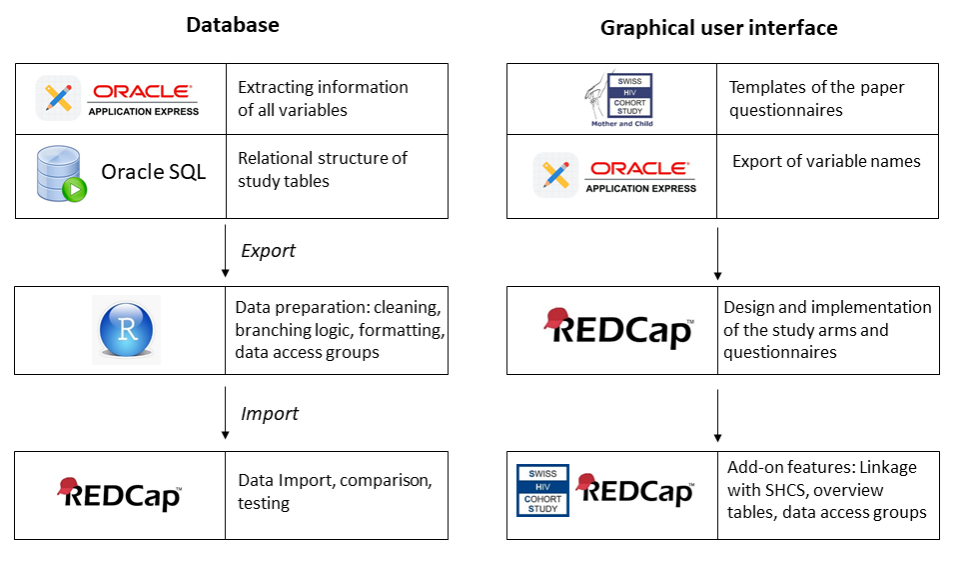
Overview of the main building blocks of the database migration, showing the main steps for migration of existing data (left) and the main steps for developing the graphical user interface (right).

The process of setting up an electronic data entry tool for MoCHiV was performed in four main steps, which are outlined in detail below (Figure 1 shows an overview). First, design: specification of the database structure and GUI was performed in REDCap. Second, data cleaning and formatting: the already collected study data were prepared to adhere to the newly defined REDCap database structure. Third, migration and compliance: the already collected study data were migrated from Oracle SQL to REDCap, with intensive testing to ensure complete compliance. Fourth, add-on features: these included the linkage between SHCS and MoCHiV, redirections between different study arms, and convenient graphical representations of study progress. For each step, specific challenges and solutions are described in Table 1.

**Table 1:** Examples of challenges faced during the migration process from Oracle SQL to REDCap^a^, proposed solutions, and the computer science background necessary to perform these tasks.

Problem descriptions			Implemented solutions		Skills needed			
**Step 1**								
	In arm 1, information about pregnancies, deliveries, and newborns is collected, with 1 unique identifier (ie, primary key) per obstetric event: the SHCS^b^ ID of the woman plus the date of the last menstrual period before the obstetric event. In the Oracle database, however, three different primary keys were used: (1) pregnancy (the SHCS ID plus the date of last menstrual period), (2) delivery (the SHCS ID plus the delivery date), and (3) newborn (the child ID). The challenge was to identify a common primary key for the 3 tables.	First, delivery dates stored in the delivery table were matched with birth dates stored in the newborn table, with matching SHCS IDs of the women. The algorithm allowed mismatches of up to 3 days that were manually reviewed. Second, the corresponding entry in the pregnancy table up to 10 months before the delivery date was matched. Missing matches were manually reviewed to identify incorrect date entries.		Implementing algorithm in R				
	In cases of twins, information about the pregnancy is the same for both children, but certain aspects concerning the delivery differ, as well as newborn information, such as the birth weight. The challenge was to collect information via a single primary key.	The delivery and newborn form in REDCap was implemented with an additional question concerning twins. Using branching logic, additional questions on the second child can be entered, keeping the same primary key. The corresponding entries in the Oracle database, that is, the separate primary keys for twins, needed to be merged.		Branching logic in REDCap, merging information in R				
**Step 2**								
	In the Oracle database, information about treatments is stored in 2 separate tables, one for the drug name and the other for changes in dosage for ongoing treatments (see “Entity-relationship diagram” in Multimedia Appendix 1). The challenge was to merge these tables in order to adhere to the specified REDCap design.	An algorithm was implemented to search dosage changes for a given treatment for a child. Lists of mismatches, such as inconsistencies in dates, were manually reviewed using the archived paper forms and by consulting the treating physician.		Implementing the algorithm in R, knowledge about HIV treatment				
	For categorical variables, a predefined list of answer choices was defined in REDCap. All categorical variables stored in the Oracle database not adhering to this predefined list of answer choices needed to be cleaned. The challenge was to clean all categorical variables in an efficient, systematic way.	For all implemented categorical variables, an SQL script was implemented to produce a list of all mismatches in the Oracle database. Lists with mismatches were corrected manually. For example, all laboratory items termed “NA” (ie, *natrium*; German for “sodium”) were deleted in a first migration attempt, as “NA” was interpreted as empty (no value available).		SQL queries				
	In the Oracle database, laboratory values such as biochemistry or hepatitis testing were stored in long format with 1 row per laboratory result combined with information on the treating physician and optional comments. To adhere to the chosen REDCap database design, the biochemistry and hepatitis values needed to be provided in long format with 1 row per laboratory date. The challenge was to merge information on the treating physician and comments into 1 field per laboratory date and patient.	Different laboratory items and values from the same day were merged. Inconsistencies for physician or comment entries, such as different physicians’ information or comments for the same laboratory date, were automatically cleaned by concatenating the information provided.		Implementing an R algorithm				
**Step 3**								
	After importing all data to REDCap, reports were created to re-export the data and compare them with the Oracle tables. The challenge was to set up comparison algorithms in a systematic, efficient way.	The number and content of entries in each Oracle table and REDCap report were compared by looping through every variable via an R Script. In cases of mismatches, the errors were analyzed and corrected (Multimedia Appendix 1, Figure S8).		Implementing an R algorithm				
**Step 4**								
	Using the REDCap functionality of data access groups, we ensured that participating physicians and study nurses could only view information on women and children enrolled at their own center. The challenge was to define these data access groups for every patient, including patients who switched centers.	Information on the last study visit of the children (information stored in MoCHiV^c^) and the women (information stored in the SHCS) were used to define the data access groups. Data needed to be cleaned for missing or inconsistent information, such as mismatched center information between different tables stored in Oracle.		REDCap functionality “Data access group” and R algorithm				

^a^REDCap: Research Electronic Data Capture

^b^SHCS: Swiss HIV Cohort Study.

^c^MoCHiV: Swiss Mother and Child HIV Cohort Study.

### Step 1: Design

**Figure 2:**
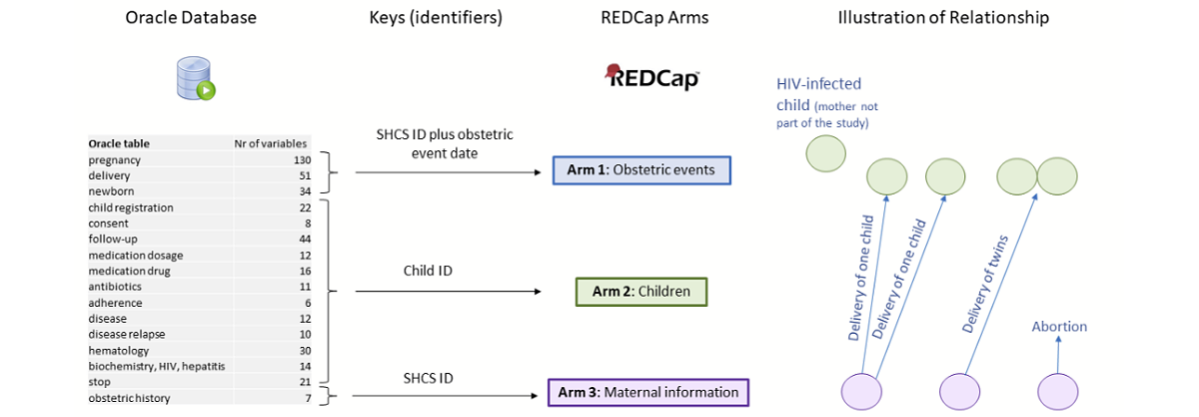
The relational structure of the Oracle database (ie, connected tables) needed to be transferred to 3 different arms in REDCap, with each arm having a different system of record identifiers (ie, primary keys). The possible relationships (eg, 0, 1, or 2 children per obstetric event and different obstetric events) are visualized. SHCS: Swiss HIV Cohort Study.

MoCHiV collects information on obstetric events including abortions among women living with HIV, longitudinal information about mother-child pairs for mothers living with HIV (eg, treatments and laboratory values), and additional in-depth information on children living with HIV. Children born to women enrolled in MoCHiV are followed up from delivery. Children living with HIV might join the study later without neonatal information and without information on the mother (eg, adopted children). To reflect these 3 different study components and relationships (ie, women, mother-child pairs, and children), 3 different study arms were designed in REDCap (see “Domains” in Multimedia Appendix 1). Figure 2 provides an overview of the available data, the mother-child-event relationship, and the chosen design in REDCap. Multimedia Appendix 1, Figure S2 shows the entity-relationship diagram for the data tables stored in Oracle SQL.

#### Arm 1: Pregnancy, Delivery, and Newborns

The primary key of this study arm, that is, the unique identifier for each obstetric event, was composed by combining the numeric SHCS identifier of the woman with the date of the obstetric event (Table 1 describes the challenge of defining obstetric event dates). Pregnancy-specific information, information on the delivery, and neonatal information can be collected in this study arm.

#### Arm 2: Child Follow-up

The primary key of this study arm was defined as the unique numeric identifier for each child. An arbitrary number of clinical visits, including laboratory values, changes in medication, or clinical events, can be stored via the repeating instrument function in REDCap, that is, by enabling a relational structure by combining the primary key (ie, child identifier) with the specific clinical visit dates.

#### Arm 3: Mother Information

The primary key of this study arm was defined as the numeric SHCS identifier of the woman. Here, baseline demographic information, as well as medication and clinical events of the women, can be reported. The 3 study arms were connected via foreign keys: the primary key of arm 3 is a foreign key of arm 1, that is, several obstetric events for the same woman can be stored, and the primary key of arm 2 is a foreign key of arm 1, that is, the child identifier (Table 1 describes the challenge of how to deal with twins).

The specific questions and answer options in each arm were designed based on the available paper forms to maintain the study content [25]. Moreover, the hierarchy and dependence of certain variables needed to be specified in REDCap; this is termed branching logic, meaning that the answers to certain questions in the questionnaire trigger different follow-up questions (see “Branching logic” in Multimedia Appendix 1).

### Step 2: Data Cleaning and Formatting

All data newly entered through the REDCap GUI automatically follows the defined REDCap database structure. Already collected data can be imported batchwise through the REDCap data import tool but need to be imported with the prespecified design developed in step 1. This includes particular valid options for categorical variables and adherence to branching logic. Otherwise, erroneous entries are not transferred to REDCap correctly or are deleted (see “Cleaning” in Multimedia Appendix 1). Since the first data entries in MoCHiV date back to 1981, the questionnaires were continuously adapted over time by including new questions, new answer options, and new branching logic (ie, dependencies in certain questions). Hence, the existing database needed extensive processing, including cleaning of erroneous and inconsistent data entries, removal of duplicated entries, and structural changes corresponding to new variable requirements. A semiautomated approach was chosen to process and clean the data. First, all data entries violating branching logic were identified in the Oracle database using SQL queries and cleaned manually (in specific cases) by going back to the paper forms or the treating physician or by cross-checking with the SHCS database. Systematic changes in data types were performed via SQL commands in the Oracle database, such as recoding answer options for categorical variables. After the cleaning procedure, the relational tables stored in the Oracle database needed to be formatted in order to adhere to the database structure of data stored in REDCap. While a REDCap instance including all its system, project, and metadata information is set up via one relational MySQL database, the newly collected data for all projects in a REDCap instance is stored in a single table, “redcap_data,” in entity-attribute-value (EAV) format. Hence, each collected data point contributes to one row in the “redcap_data” table, including the corresponding attributes of this value (see “REDCap database structure” in Multimedia Appendix 1) [26]. R was used to prepare the relational tables from Oracle SQL for the REDCap upload. This included renaming variables to obtain unique variable names throughout the whole project; defining the correct data format, including date fields; creating REDCap-specific variables; and numbering of repeated events to enable a relational data structure (see “R statistical software: data formatting” in Multimedia Appendix 1).

### Step 3: Migration and Compliance

After completing the cleaning steps described above, all tables stored in the relational MoCHiV Oracle SQL Database were exported. The R scripts for formatting were applied (see step 2 and “R statistical software: data formatting” in Multimedia Appendix 1) and the newly formatted tables were stored as comma-separated value (csv) files. The data import tool in REDCap was used to import these csv files to REDCap, which follows a 2-step import procedure. The feedback given by the import tool in the first step was analyzed (see “Data import checks in REDCap” in Multimedia Appendix 1) and in case of inconsistencies, the csv files were further adapted if inconsistencies were missed in the data cleaning and formatting phase (see step 2). This procedure was repeated for all csv files until all data points were imported without error messages in the import tool.

To ensure that no data point was lost in the migration procedure, a systematic comparison of the entries in REDCap and the Oracle database was performed. In particular, customized reports were generated using the built-in report tool to export data from REDCap in the form of csv tables for the different study arms and questionnaires. The csv files were then formatted according to the database design in Oracle SQL. Hence, the reverse step as described in the formatting part of step 2 was implemented. These csv files were then compared with the original csv files exported from the Oracle database. The number of entries and values were compared systematically using R. If there were mismatches, including data points lost or changed in the migration procedure (Table 1 shows examples), the errors were fixed, and the whole migration process was repeated. With this, we achieved a complete migration of MoCHiV data with no data points lost.

### Add-on Features

**Figure 3:**
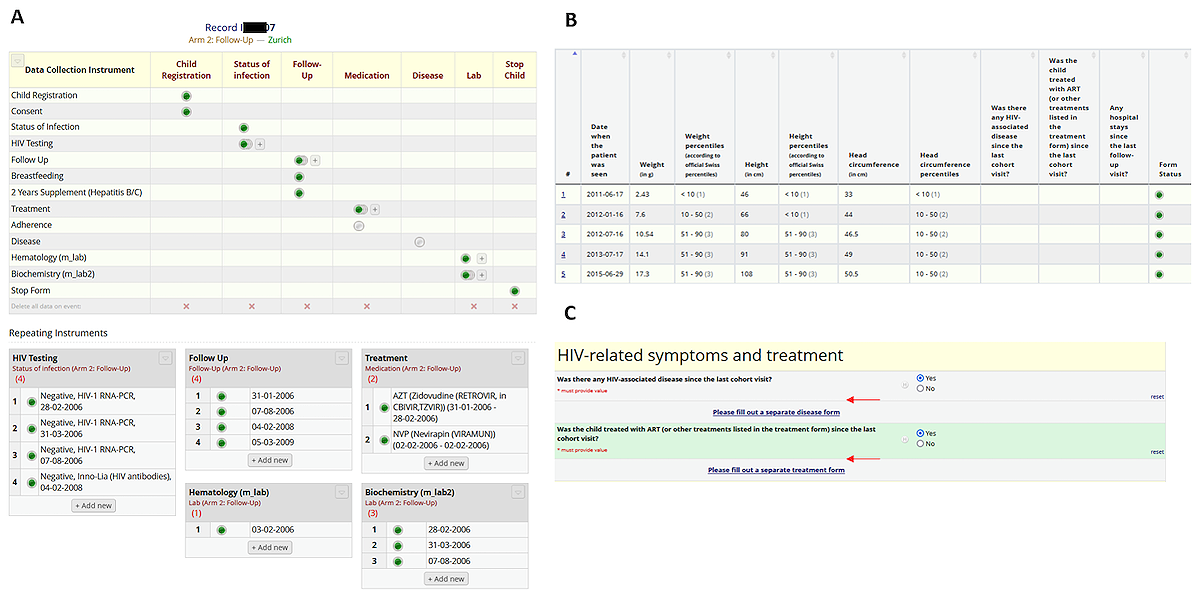
Built-in REDCap tools were used to (A) create an overview of all Swiss Mother and Child HIV Cohort Study (MoCHiV) visits of a child, (B) collect variables such as weight over time, and (C) provide intuitive redirections between different Research Electronic Data Capture (REDCap) forms.

Several built-in REDCap functions and application programming interfaces (APIs) were used to improve user-friendliness and ensure the best possible future data quality. Figure 3 highlights some of these add-on features. First, to ensure data protection in this multicenter study, REDCap data access groups were created to provide access to the different data sets to only personnel from the center where the women and children were followed up. Second, overview tables were designed in REDCap to provide convenient visualization of follow-up status, HIV testing, and medication and laboratory history of participating children. Third, to avoid duplicate data entry for women enrolled in both MoCHiV and the SHCS, information entered in the SHCS was directly linked to the SHCS database. Fourth, to increase coverage and completeness of pregnancies in MoCHiV and improve visibility of this substudy, an alert in the SHCS data management system was implemented. Fifth, a link between the child ID in MoCHiV and the SHCS ID for children transferring from MoCHiV to the SHCS at the age of 18 years was integrated to link data collected for the same individual in the 2 studies.

## Discussion

Here, we report on the successful migration of a longitudinal cohort study with a complex study design from a relational database in Oracle to REDCap. The main motivation for this database migration was to provide a user-friendly electronic data entry tool for participating study nurses and physicians. The previous paper-based system with data managers manually transcribing information was inefficient, error-prone, frequently required further inquiries by the data managers, and did not provide real-time overview of study progress. REDCap provides a management system to handle the database, provides a user-friendly GUI, and is free of charge for academic institutions (in contrast to Oracle SQL). REDCap is intuitive to use, supports flexible study designs, and offers various built-in tools for data monitoring and APIs.

Despite the convenience REDCap provides, implementing and migrating a complex study that has been ongoing for several decades is still a tremendous effort and requires additional software. The most time-consuming part of this project was cleaning, mapping, and formatting the already collected data. MoCHiV consists of over 2 million data points spread over 428 variables, more than 2000 participating children, and more than 1000 women. Although, in general, the amount of work needed to migrate a database does not necessarily scale up with a larger amount of data entries, the amount of erroneous data entries does. REDCap offers the built-in data import tool, but data still need to be provided in a predefined format. Here, programming skills for tools such as SQL and R were crucial for data cleaning and formatting. For example, REDCap-specific variables, such as indication of study arm, numbering of repeated events to enable a relational structure, and the creation of unique variable names throughout the project required extensive preprocessing. As the REDCap data import tool only provides feedback on erroneous entries and does not allow interactive data correction, all inconsistent data points and violations of branching logic needed to be corrected before uploading the data.

To summarize, starting projects from scratch in REDCap, even ones with a complicated longitudinal study design like that of MoCHiV, is feasible for data managers without programming or database knowledge, but migrating existing data to REDCap is more complicated and requires additional software. Workflows to migrate data efficiently have been proposed by other groups [14] with the conclusion that programming knowledge is key for database migrations. To date, there is no built-in REDCap solution, such as an interactive GUI, to clean, format, and alter imported data. Our study illustrates various challenges in the migration from Oracle SQL to REDCap and suggests a possible workflow for database migration using R for data preprocessing. Our proposed workflow can be translated to other studies currently stored in relational databases such as Oracle SQL.

## Appendix

Multimedia Appendix 1 Supplementary figures, tables, and information.

## Abbreviations

API: application programming interface
csv: comma-separated value
EAV: entity-attribute-value
GUI: graphical user interface
IT: information technology
MoCHiV: Swiss Mother and Child HIV Cohort Study
REDCap: Research Electronic Data Capture
SHCS: Swiss HIV Cohort Study

## Data Availability

This project describes the migration of study data stored in the Swiss Mother and Child HIV Cohort Study (MoCHiV). The metadata of this study, including the description of variables collected in this study, are publicly available [27]. The individual-level data sets collected in MoCHiV do not fulfill the requirements for open data access due to privacy concerns and the ethical consent forms signed by the patients. Nevertheless, the data can be requested for research purposes at the MoCHiV website [28].

## References

[1] Collins FS, Varmus H (2015). A new initiative on precision medicine. N Engl J Med.

[2] Duffy DJ (2016). Problems, challenges and promises: perspectives on precision medicine. Brief Bioinform.

[3] Odone A, Buttigieg S, Ricciardi W, Azzopardi-Muscat N, Staines A (2019). Public health digitalization in Europe. Eur J Public Health.

[4] Tammaro Am, Matusiak Kk, Sposito Fa, Casarosa V (2019). Data curator's roles and responsibilities: an international perspective. Libri.

[5] Campbell D (2008). Don't forget people and specimens that make the database. Nature.

[6] Spichtinger D (2022). Data management plans in Horizon 2020: what beneficiaries think and what we can learn from their experience. Open Res Europe.

[7] (2018). Everyone needs a data-management plan. Nature.

[8] Harris PA, Taylor R, Thielke R, Payne J, Gonzalez N, Conde JG (2009). Research electronic data capture (REDCap)--a metadata-driven methodology and workflow process for providing translational research informatics support. J Biomed Inform.

[9] Maré Irma Adele, Kramer B, Hazelhurst S, Nhlapho MD, Zent R, Harris PA, Klipin M (2022). Electronic Data Capture System (REDCap) for health care research and training in a resource-constrained environment: technology adoption case study. JMIR Med Inform.

[10] Odukoya O, Nenrot D, Adelabu H, Katam N, Christian E, Holl J, Okonkwo A, Kocherginsky M, Kim K, Akanmu S, Abdulkareem FB, Anorlu R, Musa J, Lesi O, Hawkins C, Okeke O, Adeyemo WL, Sagay S, Murphy R, Hou L, Ogunsola FT, Wehbe FH (2021). Application of the research electronic data capture (REDCap) system in a low- and middle income country- experiences, lessons, and challenges. Health Technol (Berl).

[11] Tran V, Gwenzi F, Marongwe P, Rutsito O, Chatikobo P, Murenje V, Hove J, Munyaradzi T, Rogers Z, Tshimanga M, Sidile-Chitimbire V, Xaba S, Ncube G, Masimba L, Makunike-Chikwinya B, Holec M, Barnhart S, Weiner B, Feldacker C (2022). REDCap mobile data collection: Using implementation science to explore the potential and pitfalls of a digital health tool in routine voluntary medical male circumcision outreach settings in Zimbabwe. Digit Health.

[12] Cheng A, Duda S, Taylor R, Delacqua F, Lewis A, Bosler T, Johnson K, Harris P (2021). REDCap on FHIR: clinical data interoperability services. J Biomed Inform.

[13] Rovera G, Fariselli P, Deandreis D (2022). Development of a REDCap-based workflow for high-volume relational data analysis on real-time data in a medical department using open source software. Comput Methods Programs Biomed.

[14] Dunn WD, Cobb J, Levey AI, Gutman DA (2016). REDLetr: Workflow and tools to support the migration of legacy clinical data capture systems to REDCap. Int J Med Inform.

[15] Girani E, Gabetta M, Alloni A, Stuppia M, Sacchi L, Barbarini N (2021). Automatic data transfer from OMOP-CDM to REDCap: A semantically-enriched framework. Volume 287: Applying the FAIR Principles to Accelerate Health Research in Europe in the Post COVID-19 Era.

[16] Shalhout S, Saqlain F, Wright K, Akinyemi O, Miller D (2022). Generalizable EHR-R-REDCap pipeline for a national multi-institutional rare tumor patient registry. JAMIA Open.

[17] Brix T, Greulich L, Janssen A (2022). Linking EMR data to REDCap: implementation in the SOLKID Register. Volume 294: Challenges of Trustable AI and Added-Value on Health.

[18] Hawley S, Yu J, Bogetic N, Potapova N, Wakefield C, Thompson M, Kloiber S, Hill S, Jankowicz D, Rotenberg D (2021). Digitization of measurement-based care pathways in mental health through REDCap and electronic health record integration: development and usability study. J Med Internet Res.

[19] Scherrer A, Traytel A, Braun D, Calmy Alexandra, Battegay Manuel, Cavassini Matthias, Furrer Hansjakob, Schmid Patrick, Bernasconi Enos, Stoeckle Marcel, Kahlert Christian, Trkola Alexandra, Kouyos Roger D, Tarr Philip, Marzolini Catia, Wandeler Gilles, Fellay Jacques, Bucher Heiner, Yerly Sabine, Suter Franziska, Hirsch Hans, Huber Michael, Dollenmaier Günter, Perreau Matthieu, Martinetti Gladys, Rauch Andri, Günthard Huldrych F, Swiss HIV Cohort Study (SHCS) (2022). Cohort profile update: the Swiss HIV Cohort Study (SHCS). Int J Epidemiol.

[20] Compagno F, Naegele K, Kahlert CR, Hösli Irene, Aebi-Popp K, Martinez de Tejada B, Paioni P, Yerly S, Böni Jürg, Battegay M, Rudin C, Hirsch HH, Swiss Hiv Cohort Study (2019). The rate of mother-to-child transmission of antiretroviral drug-resistant HIV strains is low in the Swiss Mother and Child HIV Cohort Study. Swiss Med Wkly.

[21] Aebi-Popp K, Kouyos R, Bertisch B, Staehelin C, Hoesli I, Rickenbach M, Thorne C, Grawe C, Bernasconi E, Cavassini M, de Tejada Begona Martinez, Stoeckle M, Lecompte T, Rudin C, Fehr J (2014). Loss to follow-up of HIV-infected women after delivery: The Swiss HIV Cohort Study and the Swiss Mother and Child HIV Cohort Study. J Int AIDS Soc.

[22] TESTER ALT MoCHiV key data (figures). Swiss HIV Cohort Study.

[23] Ethic committee approval and informed consent. Swiss HIV Cohort Study.

[24] Oracle APEX. Oracle.

[25] MoCHiV forms. Swiss HIV Cohort Study.

[26] Nadkarni PM, Brandt C (1998). Data extraction and ad hoc query of an entity-attribute-value database. J Am Med Inform Assoc.

[27] Forms. Swiss HIV Cohort Study.

[28] Open data statement SHCS. Swiss HIV Cohort Study.

