# Supplementary material for: Migrating a Well-Established Longitudinal Cohort Database From Oracle SQL to Research Electronic Data Entry (REDCap): Data Management Research and Design Study
Source: JMIR Form Res. 2023 May 31;7:e44567. doi: 10.2196/44567 (PMC10267784; doi:10.2196/44567)
Supplement: Multimedia Appendix 1 [file formative_v7i1e44567_app1.docx]

***Supplementary Material: Migrating a well-established longitudinal cohort database from Oracle SQL to Research Electronic Data entry (REDCap): Data Management Research and Design***

Kusejko et al.

**S1) Domains**

The REDCap instance of the Swiss HIV Cohort Study (SHCS) has the domain <https://redcap.shcs.ch/>. MoCHiV data is collected in project 46, in three different study arms:

Pregnancy, Delivery & Newborn: <https://redcap.shcs.ch/redcap_v12.5.10/DataEntry/record_status_dashboard.php?pid=46&arm=1>

Follow-up: <https://redcap.shcs.ch/redcap_v12.5.10/DataEntry/record_status_dashboard.php?pid=46&arm=2>

Maternal Information: <https://redcap.shcs.ch/redcap_v12.5.10/DataEntry/record_status_dashboard.php?pid=46&arm=3>

**S2) Entity relationship diagram**

MoChiV data was stored in an Oracle SQL database with several relational data tables. The following figure visualizes the relational database structure.


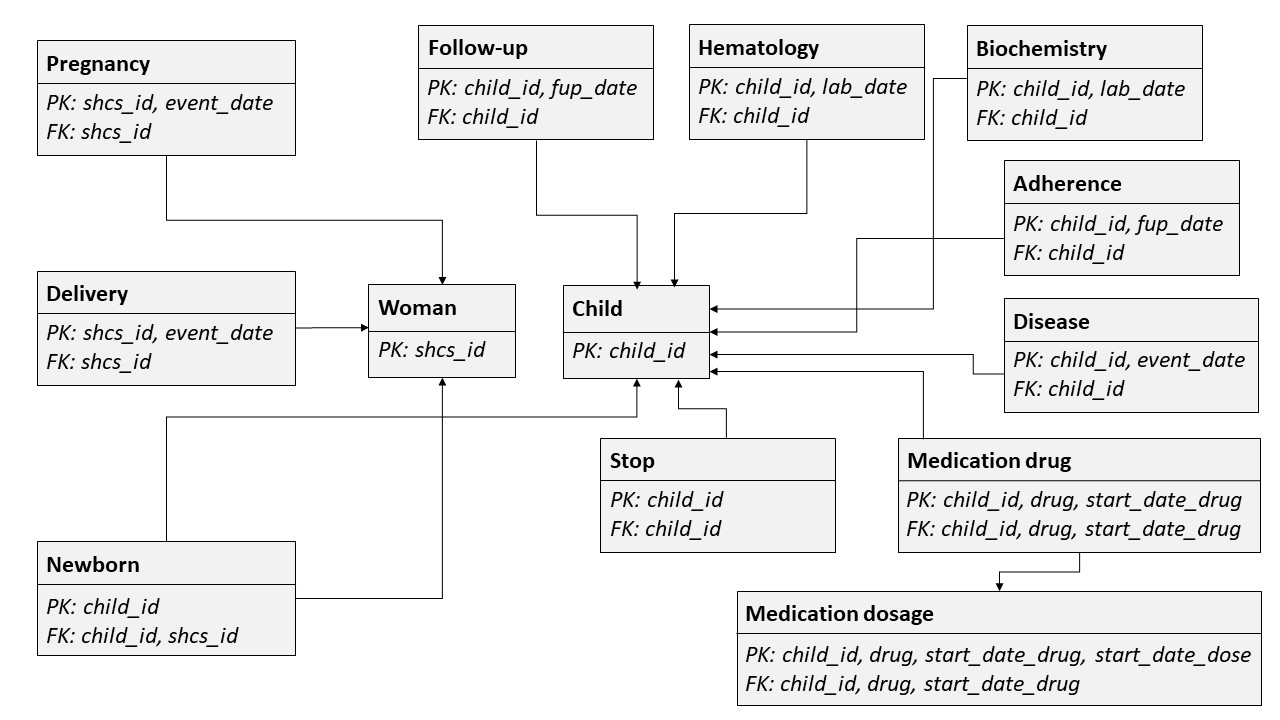


***Figure S1****: Entity-relationship diagram of the data tables stored in the Oracle SQL database. PK: Primary key, FK: Foreign key.*

**S3) Branching Logic**

In REDCap, fields can be hidden conditional on answers of previous questions. For example, the answer “no” to the question whether minor complications were experienced post-partum would hide the questions on the specific type of complications, while “yes” would make these questions visible (see **Figure S2 and S3**)


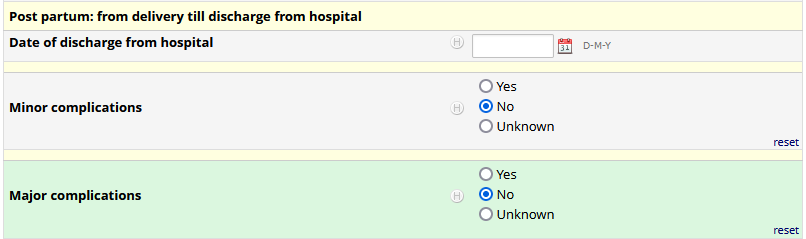


***Figure S2****: No answer options for minor complications.*


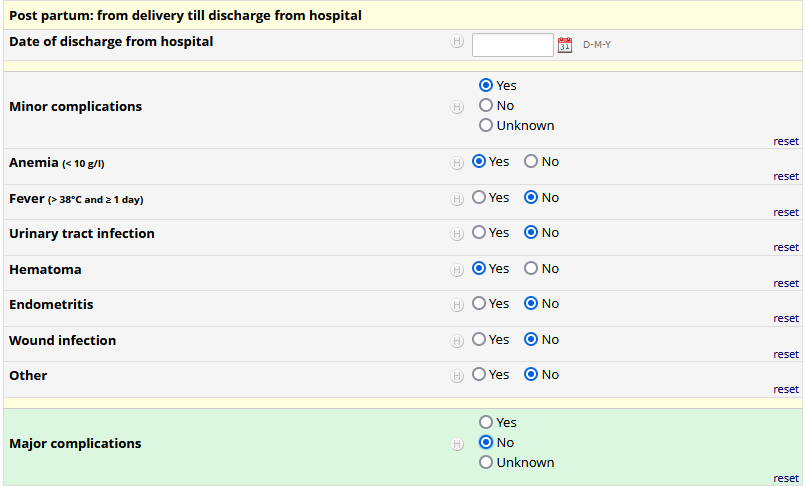


***Figure S3****: Various answer options for minor complications*

Of note, in case of violation of branching logic, e.g., changing the answer to “Minor complications” back to “no” after having filled out specific complications, REDCap offers to delete these previously entered values (see also **Section S6**, as this poses a challenge for data migration).

**S4) Cleaning**

In case of violation of the branching logic – i.e., providing answers for hidden fields – REDCap will upload the data, but prompt an error message when opening this record in the GUI. For example, in case a post-partum complication such as “fever” is given, but the question on complications is answered with “no” (see **S3, Branching Logic**), the user will be asked whether to delete this data point or not (See **Figure S4**).


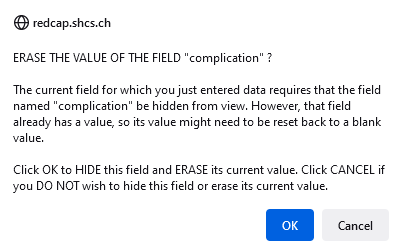


***S4:*** *Error prompt when opening a record that violates the branching logic in a question.*

To avoid the situation that study nurses and physicians will have to deal with these error messages (and might delete data), the violations of the branching logic were tested and cleaned before. For this, every branching logic was translated to an SQL statement producing the cases of violations of branching logic of data stored in the Oracle SQL database. For example, the following SQL statement in the Oracle SQL developer would yield the list of violations of the branching logic concerning minor complications (see S2 above).


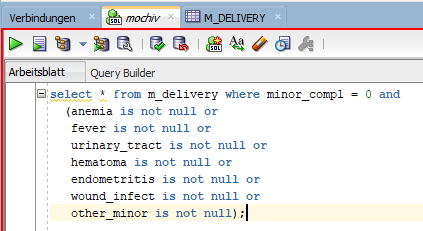


***S5:*** *Using SQL statements to produce lists of violations of the branching logic in the already collected data stored in the Oracle SQL database.*

As a result, in the final version of the data transferred to REDCap, no violation of any branching logic was present.

Further cleaning steps included:

- Deleting duplicated data entries
- Correcting contradicting information for the same participant on the same date (e.g. laboratory values)
- Defining values for missing variables which are mandatory, e.g., obstetric event date
- Correcting undefined answer choices for categorical variables (see **Section S7**)

**S5) REDCap Database Structure**

REDCap stores all data, project and system information within one single MySQL database. This means, that there is one database per REDCap instance (such as the REDCap instance of the Swiss HIV Cohort Study), with multiple studies running on this instance. The relevant information is stored in relational database tables, including tables about user information, project metadata, and the data newly collected through REDCap questionnaires (see **Figure S5** for an example of REDCap database tables). This structure implies that newly collected data – such as all study data collected in the MoCHiV study – is stored in one (non-relational) table, “redcap_data”.


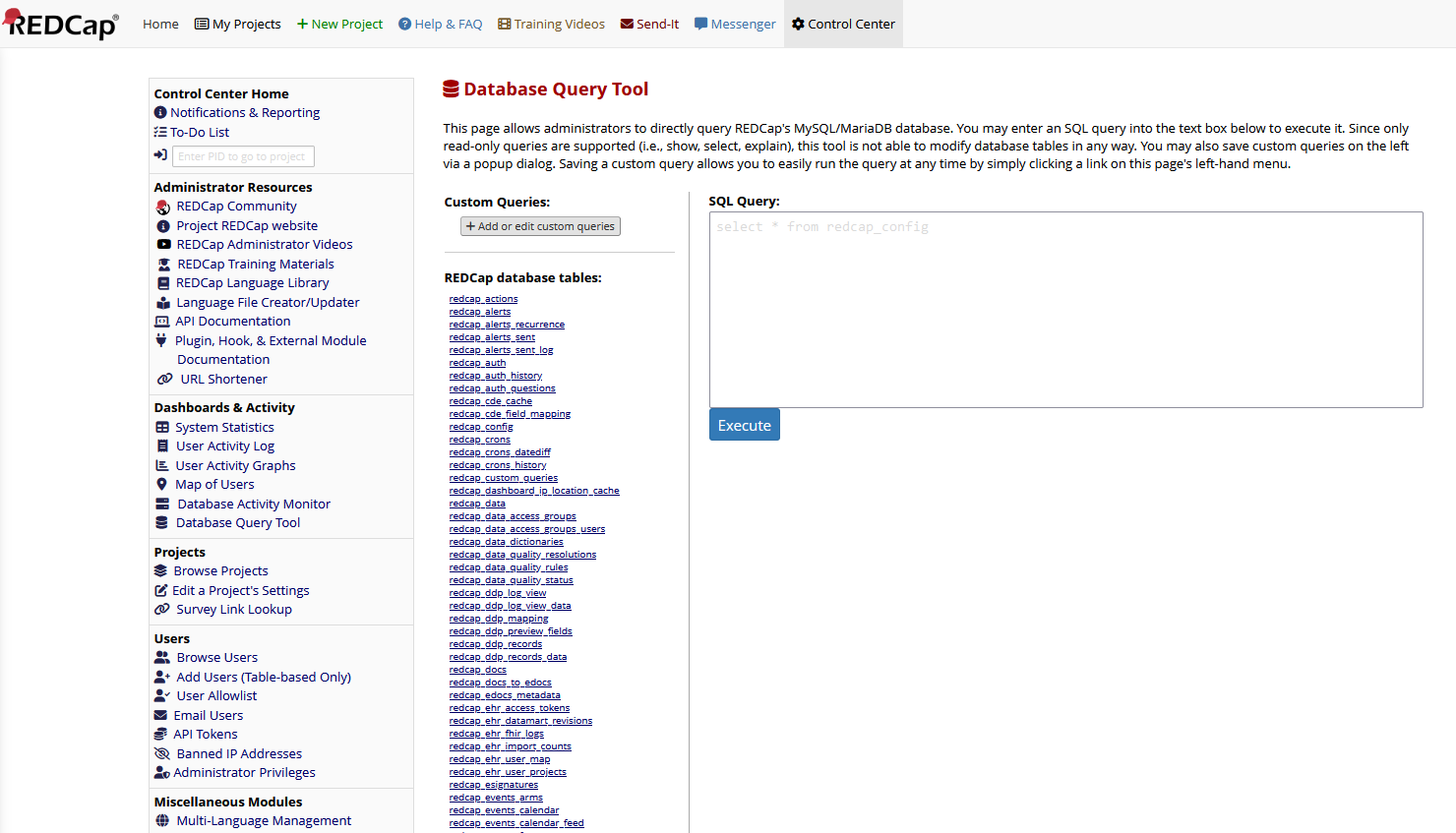


***Figure S5****: In the REDCap Control Center, all REDCap database tables stored in the MySQL database are listed.*

Study data stored in the “redcap_data” table includes information on (see **Figure S6** for an example):

- *Project ID*: Number of the project, such as 46 for the MoCHiV study on the SHCS REDCap instance
- *Event ID*: Identifier of the REDCap questionnaire, such as 154 corresponding to the laboratory information.
- *Record*: Identifier of the study subject, e.g., child identifier
- *Field Name*: The specific data entry field, such as “leu” for leucocyte counts in our MoCHiV project.
- *Value*: The data entered in the REDCap questionnaire, such as the laboratory value requested in the leucocyte variable.
- *Instance*: Since more than one data point per field can be collected - e.g., several leucocyte counts for the same study subject over time – this column keeps track of the longitudinal data entry, i.e., the many-to-one relationship.


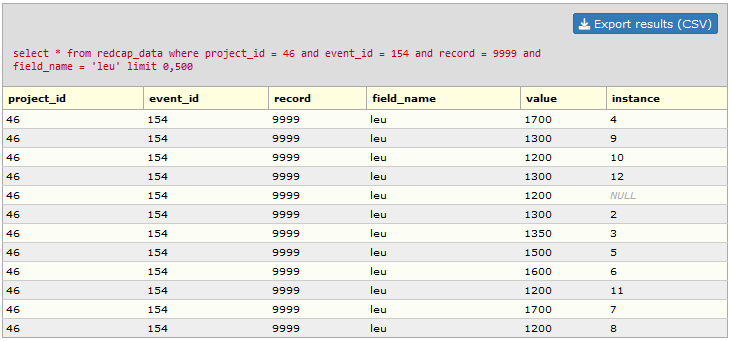


***Figure S6****: Example of leucocyte counts stored for subject 9999 (child identifier) in 12 different laboratory visits.*

Enabling the option of having repeated instruments, i.e., collecting the same information at different time points (such as leucocyte counts in the example above), includes the instance-information in the ‘redcap_data’ table, and hence allows to reconstruct the relational many-to-one relationship.

**S6) R Statistical Software: Data formatting**

Data stored in the Oracle SQL database needs to be formatted to adhere to the newly specific REDCap design. This includes in particular adding information on study arms, instruments, and numbering of repeated events. Let “df” be the name of one table downloaded from the Oracle SQL, such as the table containing all laboratory values for the children enrolled in MoCHiV. The following steps needed to be performed with R before importing the data to REDCap.

A) Unique variable names: Since the data is stored in the relational Oracle SQL database, the columns of different tables might have the same name, such as the columns “comments”, “inputdate”, “physician”, being present in all tables. REDCap does not store the study data in relational tables, and hence all variables need to have unique names. The columns needed to be renamed, as for example renaming these columns in the lab table:

| names(df)[which(names(df) == 'comments')] <- 'comments_m_lab'  names(df)[which(names(df) == 'inputdate')] <- 'inputdate_m_lab'  names(df)[which(names(df) == 'physician')] <- 'physician_m_lab' |
| --- |

B) Variable format: Data imported to REDCap needs to adhere the pre-specified data format, such as the correct format for date variables, e.g., dd.mm.yy, or the specification of character of integer format.

| col_types = cols(LABDATE = col_date(format = "%d.%m.%y"),  CD4DATE = col_date(format = "%d.%m.%y"),  COMMENTS = col_character(),  INPUTDATE = col_date(format = "%d.%m.%y"), … |
| --- |

C) REDCap-specific variables: When importing data to REDCap, the event and instrument needs to be specified. For example, when importing the laboratory values (hematology), the following variables need to be created:

| df <- cbind.data.frame(df,  redcap_event_name = 'lab_arm_2',  redcap_repeat_instrument = 'hematology_m_lab',  hematology_m_lab_complete = 2) |
| --- |

The “complete” variables ensures that the data points are considered as completed (indicated by a green color in the REDCap GUI, otherwise the color would be red or yellow).

D) Enabling relational structure: To store multiple data points for the same record ID, for example multiple platelet counts for the same child on different dates, the repeated instrument function needs to be enabled. The data points are stored in REDCap by numerating them in the variable “redcap_repeat_instance”. This numbering was not needed previously in the relational database in REDCap and hence this variable needs to be generated before importing the data. The following R-Code assigns ascending numbers to the laboratory information for the children (unique record_id per child):

| df <- df %>% group_by(record_id) %>%  arrange(labdate, .by_group = TRUE) %>% mutate(redcap_repeat_instance = 1:n()) |
| --- |

**S7) Data Import Checks in REDCap**

The REDCap Import Tool uses a two-step procedure. First, the data is checked concerning inconsistencies with the pre-defined design and for duplicates, second, the data is imported.

For example, in case of invalid answer choices, an error message would be prompted prohibiting upload of this record (see **Figure S7**).


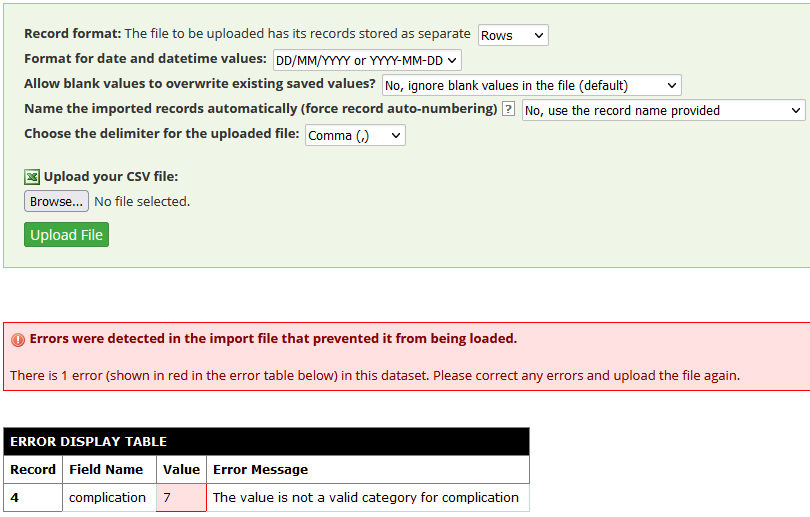


***Figure S7****: The question on complications allows the following answer choices: 0: no, 1: yes, 9: unknown; In case the cvs file for upload contains the value 7, i.e., not defined answer choice, an error message is prompted in the REDCap Import Tool.*

In case already uploaded data would be affected by changes – i.e., over-writing existing data – this is indicated with red brackets (see Figure S8).


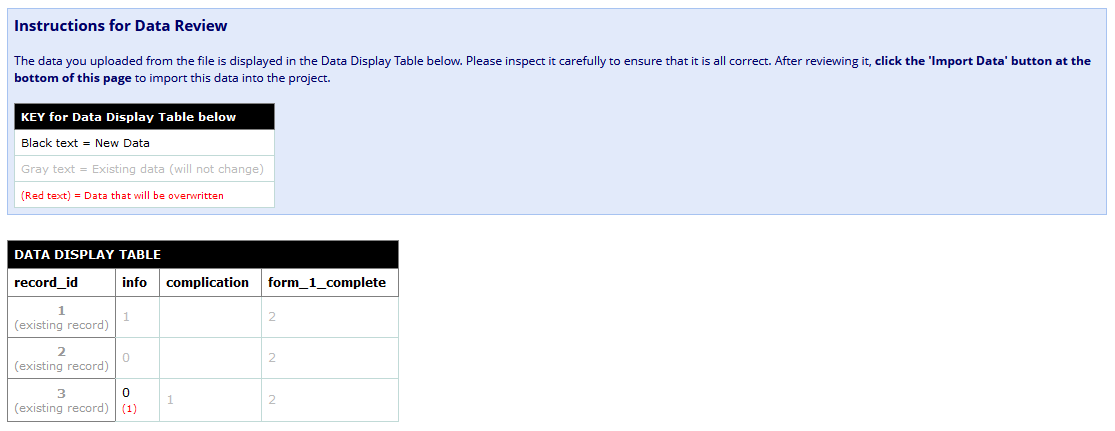


***Figure S8****: Feedback provided by the REDCap Import Tool: All three uploaded data sets already exist, with a change in variable “info” in record 3. This value will be overwritten when importing the data.*

In general, new records are indicated with “(new record)” in green brackets (see **Figure S9**).


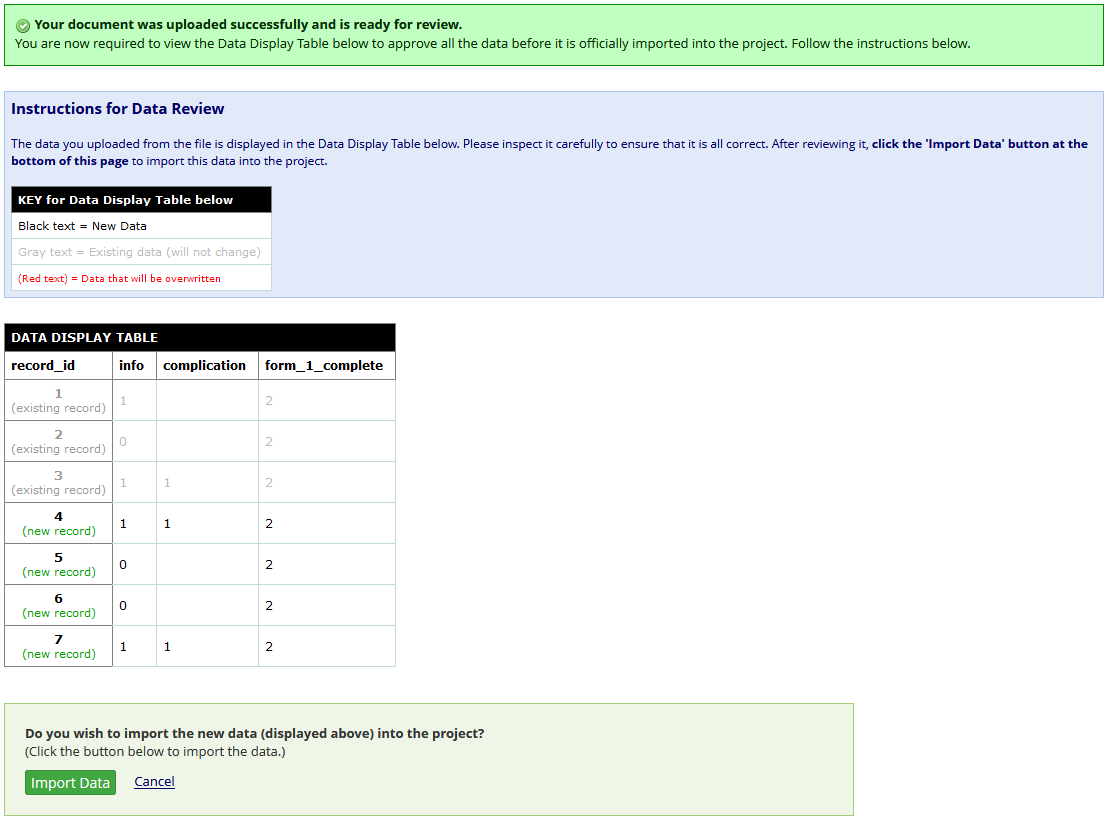


***Figure S9****: Feedback provided by the REDCap Import Tool: New records and already existing records.*
